# The effects of tributyrin supplementation on weight gain and intestinal gene expression in broiler chickens during *Eimeria maxima*-induced coccidiosis^1^

**DOI:** 10.1016/j.psj.2021.01.007

**Published:** 2021-01-18

**Authors:** Victoria L. Hansen, Stanislaw Kahl, Monika Proszkowiec-Weglarz, Stephanie C. Jiménez, Stefan F.C. Vaessen, Lori L. Schreier, Mark C. Jenkins, Beverly Russell, Katarzyna B. Miska

**Affiliations:** ∗ Animal Biosciences and Biotechnology Laboratory, Henry A. Wallace Beltsville Agricultural Research Center, Beltsville, MD 20705, USA; † Perstorp, 211 20 Malmo, Sweden; ‡ United States Department of Agriculture, Agricultural Research Service, Animal Parasitic Diseases Laboratory, Beltsville, MD 20705, USA

**Keywords:** butyrate, coccidia, broiler chickens, intestine, gene expression

## Abstract

Butyrate is a feed additive that has been shown to have antibacterial properties and improve gut health in broilers. Here, we examined the performance and gene expression changes in the ileum of tributyrin–supplemented broilers infected with coccidia. Ninety-six, Ross 708 broilers were fed either a control corn–soybean–based diet (**−BE**) or a diet supplemented with 0.25% (w/w) tributyrin (**+BE**). Birds were further divided into groups that were inoculated with *Eimeria maxima* oocysts (**EM**) or sham-inoculated (**C**) on day 21 posthatch. At 7 d postinfection (**7 d PI**), the peak of pathology in *E. maxima* infection, tributyrin-supplemented birds had significantly improved feed conversion ratios (**FCR**, *P* < 0.05) and body weight gain (**BWG**, *P* < 0.05) compared with -BE-infected birds, despite both groups having similar feed intake (**FI**, *P* > 0.05). However, at 10 d post-infection (**10 d PI**) no significant effects of feed type or infection were observed. Gene expression in the ileum was examined for insights into possible effects of infection and tributyrin supplementation on genes encoding proteins related to immunity, digestion, and gut barrier integrity. Among immune-related genes examined, *IL-1B* and *LEAP2* were only significantly affected at 7 d PI. Transcription of genes related to digestion (*APN*, *MCT1*, *FABP2*, and *MUC2*) were primarily influenced by infection at 7 d PI and tributyrin supplementation (*FABP2* and *MUC2*) at 10 d PI. With exception of *ZO1,* tight junction genes were affected by either infection or feed type at 7 d PI. At 10 d PI, only *CLDN1* was not affected by either infection or feed type. Overall tributyrin shows promise as a supplement to improve performance during coccidiosis in broiler chickens; however, its effect on gene expression and mode of action requires further research.

## Introduction

Coccidiosis in chickens is a persistent problem in poultry production that is thought to cost the poultry industry billions of dollars annually; however, its true cost is difficult to calculate (Williams, 1999; Dalloul and Lillehoj, 2006; Rushton and Bruce, 2017). Coccidiosis impacts the broiler industry because the infection results in loss of weight gain even when it does not result in bird mortality (Williams, 1999; Shirley et al., 2007). Seven defined species of *Eimeria* are known to infect chickens and are ubiquitous worldwide (Lillehoj et al., 2015). As the demand for chicken as a source of protein expands globally, so does the need to control coccidiosis in commercial production facilities.

*Eimeria* infects chickens via the fecal-oral route, and each species invades epithelial cells in specific portions of the intestine (Chapman, 2014; Lillehoj et al., 2015). *Eimeria* sporozoites invade the epithelium of the intestine, and the subsequent asexual and sexual reproduction of the parasite results in cell damage, diarrhea, possible secondary bacterial infections, and even mortality (Lillehoj et al., 2015). In the course of the infection, nutrient uptake is impaired, thus leading to a reduction of weight gain over 5 to 7 d (Bumstead and Millard, 1987; Conway et al., 1990). Because the grow-out period for broiler chickens is typically 4 to 8 wk, the loss of weight gain during *Eimeria* infection can be substantial when taken in the context of industrial poultry production. There are numerous anticoccidial drugs available (reviewed in Peek and Landman, 2011), but the demand for antibiotic-free chicken has driven the search for therapeutic alternatives. Feed additives such as organic acids that have antimicrobial activity and show evidence of improving growth performance are an attractive alternative to in-feed antibiotics.

One such feed additive is the short-chain fatty acid (**SCFA**) butyrate, a compound naturally synthesized by gut microbiota (Sakata, 1987). Butyrate has been investigated as a therapeutic supplement for both humans and livestock to improve gut health. Butyrate has been shown to increase the rate of tight junction assembly in cultured human intestinal cells which suggests butyrate helps to maintain gut integrity (Peng et al., 2009). Another study observed increased production of mucin, a secreted component of intestinal mucosal barriers, in human intestinal epithelial cells incubated with SCFA, including butyrate (Willemsen et al., 2003). Butyrate as a feed additive for broiler chickens is known to have beneficial properties such as reducing *Salmonella* colonization and increasing antimicrobial peptide transcription (Fernández-Rubio et al., 2009; Sunkara et al., 2011). Additionally, even in healthy broilers, butyrate supplementation can improve reduced weight gain caused by a lower nutrient diet (Bortoluzzi et al., 2017).

In this study, we examined the effects of butyrate supplementation on growth performance and gene transcription during coccidiosis caused by *Eimeria maxima*. We chose to focus on *E. maxima* because it is one of the most prevalent species of coccidiosis that infects chickens (McMullin, 2008). Because unprotected butyrate is absorbed in the upper gastrointestinal tract, and broiler chickens are typically supplemented with tributyrin, a triglyceride of butyrate (Bolton and Dewar, 1965). This form of butyric acid glycerol esters ensures that the butyrate is not absorbed before reaching the small intestine because lipases secreted in the small intestine are needed to cleave the covalent ester bond (Sampugna et al., 1967; Leeson et al., 2005). Tributyrin is also more palatable for consumption than uncoupled butyrate, and therefore, feed consumption is less likely affected (Leeson et al., 2005). The aim of this study was to determine if tributyrin supplementation can improve the weight gain and feed conversion ratio of broiler chickens infected with *E. maxima*. We also examined the transcription of genes in the intestine that may be directly or indirectly affected by butyrate because gene expression patterns may shed light on performance outcomes and lead to additional future treatment targets.

## Materials and Methods

### Animal and Experimental Protocols

This experiment was approved by the Animal Care and Use Committee of the Beltsville Agricultural Research Center. Animal care and husbandry was performed as previously described (Proszkowiec-Weglarz et al., 2020). Ross 708 male broiler chicks were obtained on day of hatch from Longenecker's Hatchery (Elizabethtown, PA) and housed in 4 open top wire brooder cages (1.00 m^2^), approximately 25 birds per pen, until 18 d posthatch. On day 19 posthatch, all birds were transferred to 24 battery cages with 4 birds per cage. Birds had access to food and water ad libitum throughout the experiment. Starting at day of hatch, birds were fed with a corn–soybean–based diet (crumbles, 24% protein). The diet was supplemented either with 0 (**−BE**) or 0.25% (w/w) tributyrin, a form of butyric acid glycerol esters (**+BE**, ProPhorce SR 130, Perstorp, Waspik, Netherlands). At 21 d posthatch, the birds were weighed and infected by an oral gavage of 10^3^ *E. maxima* oocysts (**EM**) or were sham-infected with water (control, **C**). *E. maxima* oocysts used were the highly pathogenic APU1 strain previously described (Jenkins et al., 2017).

All birds were sacrificed by cervical dislocation on either 7 d postinfection (**7 d PI**) or 10 d postinfection (**10 d PI**). Birds sacrificed at 7 and 10 d PI were housed in different cages. Both birds and feed were weighed at each time point to calculate body weight gain (**BWG;** calculated by subtracting weight at d 21 from final weight at either day 7 or 10 post hatch), feed intake (**FI;** the amount of feed consumed between day 21 of age and end of study), and feed conversion ratio (**FCR;** calculated by dividing the amount of feed consumed between day 21 of age and end of study by the amount of weight gain). Following sacrifice, blood samples were collected by cardiac puncture into EDTA containing tubes. Samples were centrifuged at 2,000 × *g* at 4°C to collect plasma for carotenoid measurements using previously described methods (Allen, 1987).

Following sacrifice, 1 cm sections of the ileum from 2 birds per cage (n = 6 per treatment group) were excised and snap frozen in liquid nitrogen. Tissue samples were stored at −80°C until RNA isolation.

### RNA Isolation

RNA was extracted from 100 mg of ileum per sample using the Pure Link RNA Mini Kit according to manufacturer's instructions (ThermoFisher Scientific, Waltham, MA). RNA sample quality was assessed by RNA 6000 Nano chip (Agilent Technologies, Santa Clara, CA) on a Bioanalyzer 2100 (Agilent Technologies). RNA quantity per sample was measured by NanoDrop 2000 Spectrophotometer (ThermoFisher). RNA was stored at -80°C until cDNA synthesis.

### cDNA Synthesis

All cDNA synthesis reactions (20 μL) were performed using 0.2 μg of RNA, 0.5 mmol dNTP mix (ThermoFisher), and 2.5 nmol anchored oligo dT primers (Integrated DNA Technologies, Coralville, IA, 5′-CGGAATTCTTTTTTTTTTTTTTTTTTTTV-3′) and incubated at 65°C for 5 min and cooled to 4°C for at least 1 min. Then 40 units of RNaseOUT recombinant ribonuclease inhibitor (Invitrogen, Carlsbad, CA), 5 mM DTT (Invitrogen), 200 units of SuperScript IV reverse transcriptase (Invitrogen), and 4 μL 5x SuperScript IV buffer (Invitrogen) were added to each reaction and incubated at 50°C for 10 min, 80°C for 10 min, and cooled to 4°C for at least 1 min. A pool of all RNA (0.2 μg) from all treatment groups was used as a negative control for genomic DNA contamination and was processed as the other samples but with omission of Superscript IV enzyme. Completed cDNA synthesis reactions were diluted 10-fold using UltraPure distilled nuclease-free water (Invitrogen) and stored at −20°C until use in quantitative PCR (**qPCR**) reactions.

### qPCR Data Collection

All gene expression was evaluated by qPCR using 10 μL SsoAdvanced Universal SYBR Green Supermix (Bio-Rad Laboratories, Hercules, CA), 2 μL diluted cDNA, 5 mmol forward primer, 5 mmol reverse primer, and filled to a 20 μL reaction volume with 4 μL UltraPure distilled nuclease-free water (Invitrogen). All primer sequences can be found in Table 1. Reactions were performed in triplicate on a CFX96 Touch Real-Time PCR Detection System (Bio-Rad). The cycling parameters were as follows: 95°C for 3 m, 39 cycles of 60°C for 30 s, 70°C for 30 s. Dissociation curve analysis and gel electrophoresis were employed to ensure that a single PCR amplicon of appropriate size was amplified in each reaction.

**Table 1:** qPCR primers.

Gene	Accession number	Forward primer	Reverse primer	Amplicon size (bp)	Reference
ACTB	X0082	TTCTTTTGGCGCTTGACTCA	GCGTTCGCTCCAACATGTT	88	Proszkowiec-Weglarz et al., (2019)
GAPDH	NM_204305	AGCCATTCCTCCACCTTTGAT	AGTCCACAACACGGTTGCTGTAT	112	Proszkowiec-Weglarz et al., (2019)
B2M	Z48921	TGGAGCACGAGACCCTGAAG	TTTGCCGTCATACCCAGAAGT	161	Proszkowiec-Weglarz et al., (2019)
LEAP2	NM_001001606.1	CTCAGCCAGGTGTACTGTGCTT	CGTCATCCGCTTCAGTCTCA	66	Casterlow et al., (2011)
IL1B	NM_204524	GCATCAAGGGCTACAAGCTC	CAGGCGGTAGAAGATGAAGC	131	Adedokun et al., (2012)
IL12	FJ907696.1	GCCCCGTACTGGAAAGTTCT	GGATGTCAGCACCCTCAGAT	206	Settle et al., (2015)
APN	NM_204861	GAATACGCGCTCGAGAAAAC	CTTGTTGCCAATGGAGGAGT	195	Gilbert et al., (2007)
MCT1	NM_001006323	TCTTTGGCTTTGCCTTTGGC	GTGGTTGAGCTTACCGAGCA	158	-1
FABP2	NM_001007923	AGGCTCTTGGAACCTGGAAG	CTTGGCTTCAACTCCTTCGT	139	Proszkowiec-Weglarz et al., (2020)
MUC2	NM_001318434	AAACAACGGCCATGTTTCAT	GTGTGACACTGGTGTGCTGA	127	Proszkowiec-Weglarz et al., (2019)
OCLN	NM_205128	GATGGACAGCATCAACGACC	CTTGCTTTGGTAGTCTGGGC	142	Proszkowiec-Weglarz et al., (2020)
CLDN1	NM_001013611	GGTGAAGAAGATGCGGATGG	TCTGGTGTTAACGGGTGTGA	139	Proszkowiec-Weglarz et al., (2020)
CLDN4	AY435420	ATCGCCCTGTCCGTCATC	ACCACGCAGTTCATCCACAG	137	Proszkowiec-Weglarz et al., (2020)
JAM2	XM_015299112	CTGCTCCTCGGGTACTTGG	CCCTTTTGAAAATTTGTGCTTGC	135	Proszkowiec-Weglarz et al., (2020)
JAM3	XM_417876	CCAGAGTGTTGAGCTGTCCT	AGAATTTCTGCCCGAGTTGC	147	Proszkowiec-Weglarz et al., (2020)
ZO1	XM_015278975	GCCAACTGATGCTGAACCAA	GGGAGAGACAGGACAGGACT	141	Proszkowiec-Weglarz et al., (2020)

Abbreviations: *APN*, aminopeptidase N; *CLDN1*, claudin 1; *CLDN4*, claudin 4; *FABP2*, fatty-acid binding protein 2; *IL1B,* interleukin-1 beta; *JAM3,* junctional adhesion molecule 3; *LEAP2*, liver-enriched antimicrobial peptide 2; *MCT1,* monocarboxylate transporter 1; *MUC2*, mucin 2; *OCLN*, occluding; *ZO1*, zona occludens 1.

1 Primers designed with primer3 (http://bioinfo.ut.ee/primer3-0.4.0/primer3/); (Untergasser et al., 2007).

The qPCR data were normalized to the geometric mean (Vandesompele et al., 2002) using 3 reference genes: beta actin, glyceraldehyde 3-phosphate dehydrogenase, and beta-2-microglobulin and transformed using the equation 2^−Ct^, where Ct represents the fractional cycle number when the amount of amplified product reached a fixed threshold for fluorescence. Then, the data were analyzed and presented as fold changes relative to C-BE group.

### Statistical Analysis

Performance data (BWG, FI, and FCR) and carotenoids plasma concentration were analyzed by two-way ANOVA using the general linear models of the Statistical Analysis System software v9.4 (SAS© Institute, Cary, NC, USA). Pen was considered as experimental unit.

Gene expression data were analyzed separately on either 7 or 10 d PI by two-way ANOVA using Prism 8 (GraphPad Prism, La Jolla, CA). Feed type (−BE, +BE), infection status (C, EM), and their interaction were set as the fixed effects, with significance set at *P* < 0.05. When significant interaction between main effects was detected, Tukey's test was used for multiple comparison.

## Results

### Performance Data

**Figure 1:**
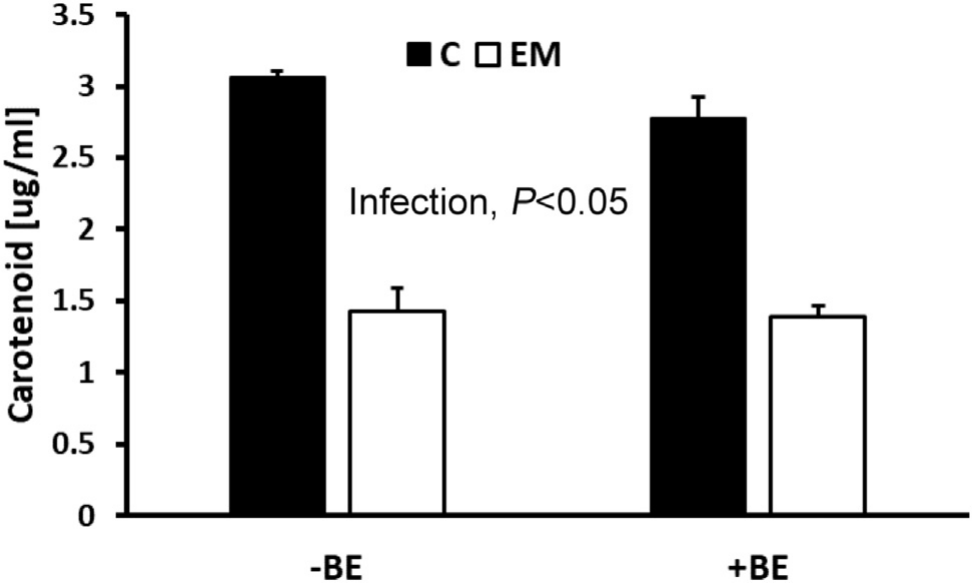
Effect of presence (+) or absence (−) of butyric acid glycerol esters (BE) in a feed on plasma carotenoid concentration in control (C) or *Eimeria maxima* (EM) challenged birds 7 d postinfection (PI). Each value represents mean ± SE (n = 6).

*E. maxima–*infected birds (BE + EM and EM) had lower (*P* < 0.05) BWG 7 d PI (Table 2), whereas no differences were observed 10 d PI (Table 2). The decrease in BWG was significantly (*P* < 0.05) attenuated in birds receiving BE (BE + EM vs. EM) 7 d PI. No effects (*P* > 0.05) of BE or EM on FI were observed at 7 and 10 d PI (Table 2). The FCR was higher (*P* < 0.05) in birds infected with EM but not supplemented with BE in their feed (EM) 7 d PI (Table 2), whereas at day 10 PI, infected birds had higher (*P* < 0.05) FCR regardless of BE supplementation (Table 2). Plasma carotenoid levels of EM infected birds were decreased (*P* < 0.05) at 7 d PI regardless of whether the diet was supplemented with BE (Figure 1).

**Table 2:** Mean ± SE of body weight gain (BWG), feed intake (FI), and feed conversion ratio (FCR) in 7 and 10 d postinfection (d PI) broilers either sham-inoculated (C) or infected with *Eimeria maxima* oocysts (EM) while eating normal feed (−BE) or feed supplemented with tributyrin (+BE).

Growth parameters	Time	C	C + BE	EM	EM + BE
BWG (g/pen)	7 d PI	2,113.7 ± 59.5^a^	1,920.3 ± 1,64.1^a^	1,230.3 ± 78.2^c^	1,568.0 ± 22.4^b^
	10 d PI	2,738.0 ± 263.4	2,585.7 ± 2,47.8	1,943.3 ± 346.3	2,331.3 ± 59.5
FI (g/pen)	7 d PI	3,264.3 ± 192	3,205.7 ± 184.4	3,335.0 ± 102.1	3,102.3 ± 113.5
	10 d PI	4,712.3 ± 696.7	4,359.0 ± 213.2	4,840.0 ± 354.7	4,719.0 ± 36.7
FCR (g Feed/g BWG)	7 d PI	1.54 ± 0.06^b^	1.69 ± 0.15^b^	2.74 ± 0.24^a^	1.98 ± 0.07^b^
	10 d PI	1.70 ± 0.10^b^	1.70 ± 0.09^b^	2.60 ± 0.32^a^	2.07 ± 0.21^a^

Different letters denote statistically significant (*P* < 0.05) differences for mean comparison between treatment groups in a single row.

### Immune-Related Genes

**Figure 2:**
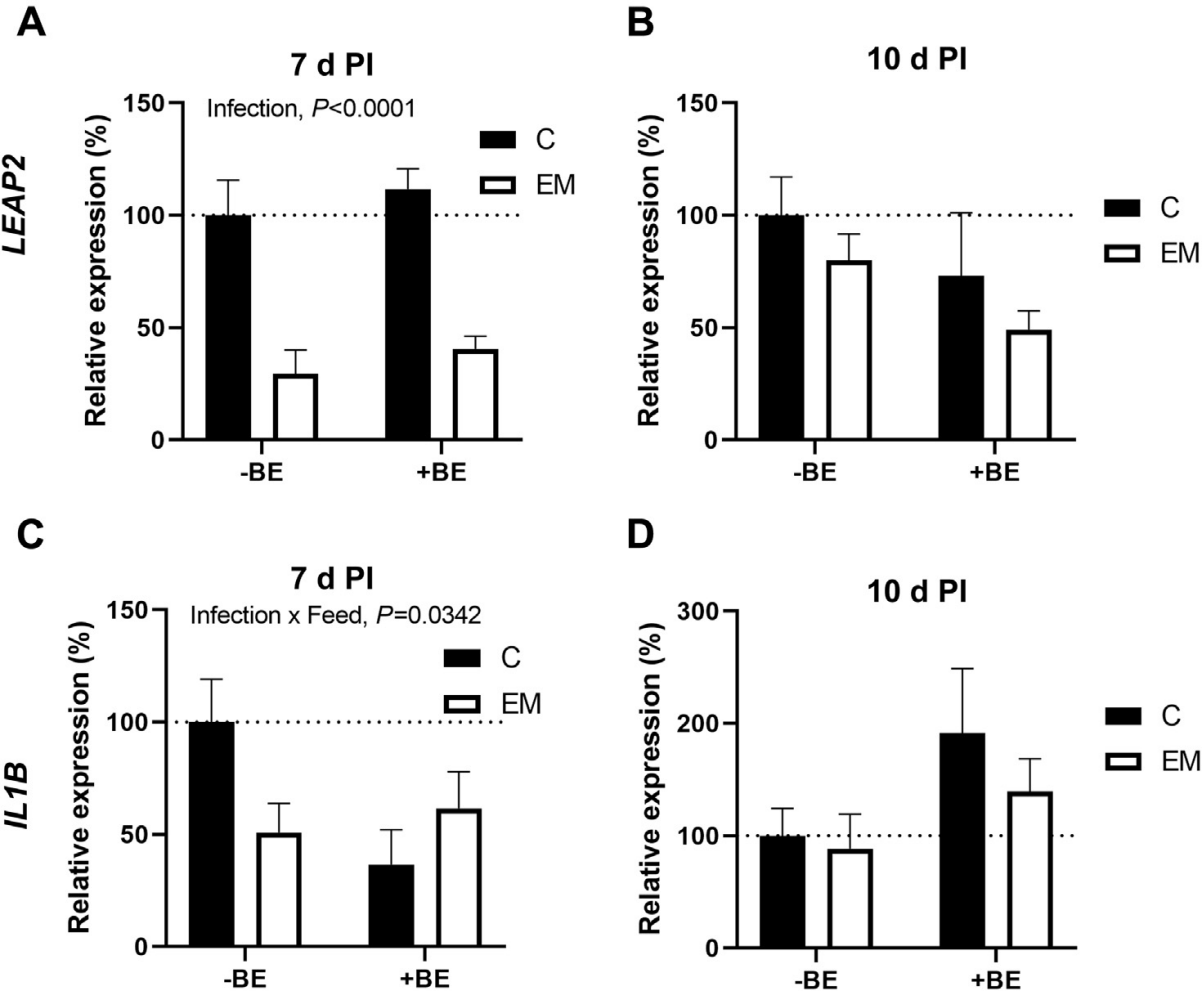
Effect of presence (+BE) or absence (−BE) of tributyrin in a feed for control (C) or *E. maxima* (EM)–challenged birds on *LEAP2* (A, B) and *IL1B* (C, D) mRNA level in chicken ileum at either 7 (7 d PI) or 10 d postinfection (10 d PI). The expression level of C-BE was set to 100% (dotted line), and the other values are presented as % of the C-BE data. Each value represents mean ± SE (n = 6). Abbreviations: *IL1B,* interleukin-1 beta; *LEAP2*, liver-enriched antimicrobial peptide 2.

The relative mRNA expression levels of liver-enriched antimicrobial peptide 2 (***LEAP2***) and interleukin-1 beta (***IL1B***) are shown in Figure 2. Interleukin-12 (***IL12***) was tested as well but has not been included here because of a lack of significant differences between groups (data not shown).

Two-way ANOVA revealed that infection (*P* < 0.0001) had a significant impact on *LEAP2* expression at 7 d PI with lower mRNA levels in EM groups regardless of feed type (Figure 2A). At 10 d PI, neither infection nor feed had a significant impact on *LEAP2* mRNA levels, but there was a trend toward lower *LEAP2* levels (*P* = 0.0719) in +BE groups (Figure 2B).

Interleukin-1 beta at 7 d PI was the only gene we tested that had a significant interaction of infection and feed (*P* = 0.0342) though Tukey's test did not find significant differences between groups. The C-BE group had the highest *IL1B* mRNA levels, and all other compared groups had means ranging between 36 and 61% (Figure 2C). At 10 d PI, there was no significant effects of infection, but there was a trend in +BE groups having higher expression than in −BE groups (*P* = 0.0525, Figure 2D).

### Enzyme, Nutrient Transporter, and Mucosal Barrier Genes

**Figure 3:**
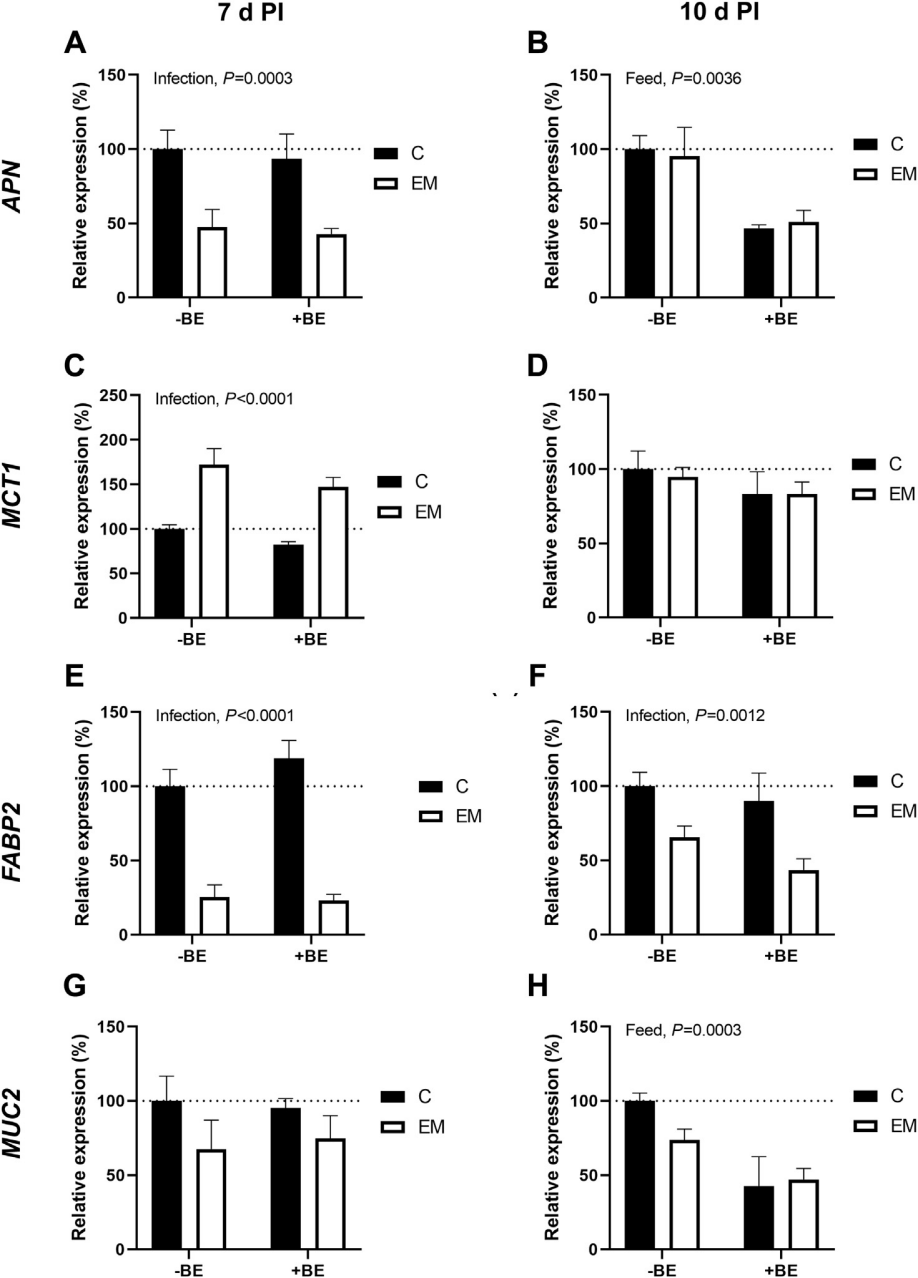
Effect of presence (+BE) or absence (−BE) of tributyrin in a feed for control (C) or *E. maxima* (EM)–challenged birds on *APN*, *MCT1*, *FABP2*, and *MUC2* mRNA level in chicken ileum at either 7 (7 d PI) or 10 d postinfection (10 d PI). The expression level of C-BE was set to 100% (dotted line), and the other values are presented as % of the C-BE data. Each value represents mean ± SE (n = 6). Abbreviations: *APN*, aminopeptidase N; *FABP2*, fatty-acid binding protein 2; *MCT1,* monocarboxylate transporter 1; *MUC2*, mucin 2.

The relative mRNA expression of aminopeptidase N (***APN***), monocarboxylate transporter 1 (***MCT1***), fatty-acid binding protein 2 (***FABP2***), and mucin 2 (***MUC2***) can be found in Figure 3. According to two-way ANOVA results, the relative expression of *APN* at 7 d PI was significantly influenced by infection (*P* = 0.0003). The EM groups had lower *APN* mRNA levels than C groups (Figure 3A). At 10 d PI, feed (*P* = 0.0036) had a significant effect on *APN* mRNA levels. The +BE groups had lower APN mRNA levels than -BE groups (Figure 3B).

For *MCT1* at 7 d PI, there were significant effects of infection (*P* < 0.0001) on mRNA levels. Broiler chickens in the EM groups had higher *MCT1* expression at 7 d PI (Figure 3C). At 10 d PI, there were no significant effects on MCT1, and no groups had significantly different mRNA levels (Figure 3D).

**Figure 4:**
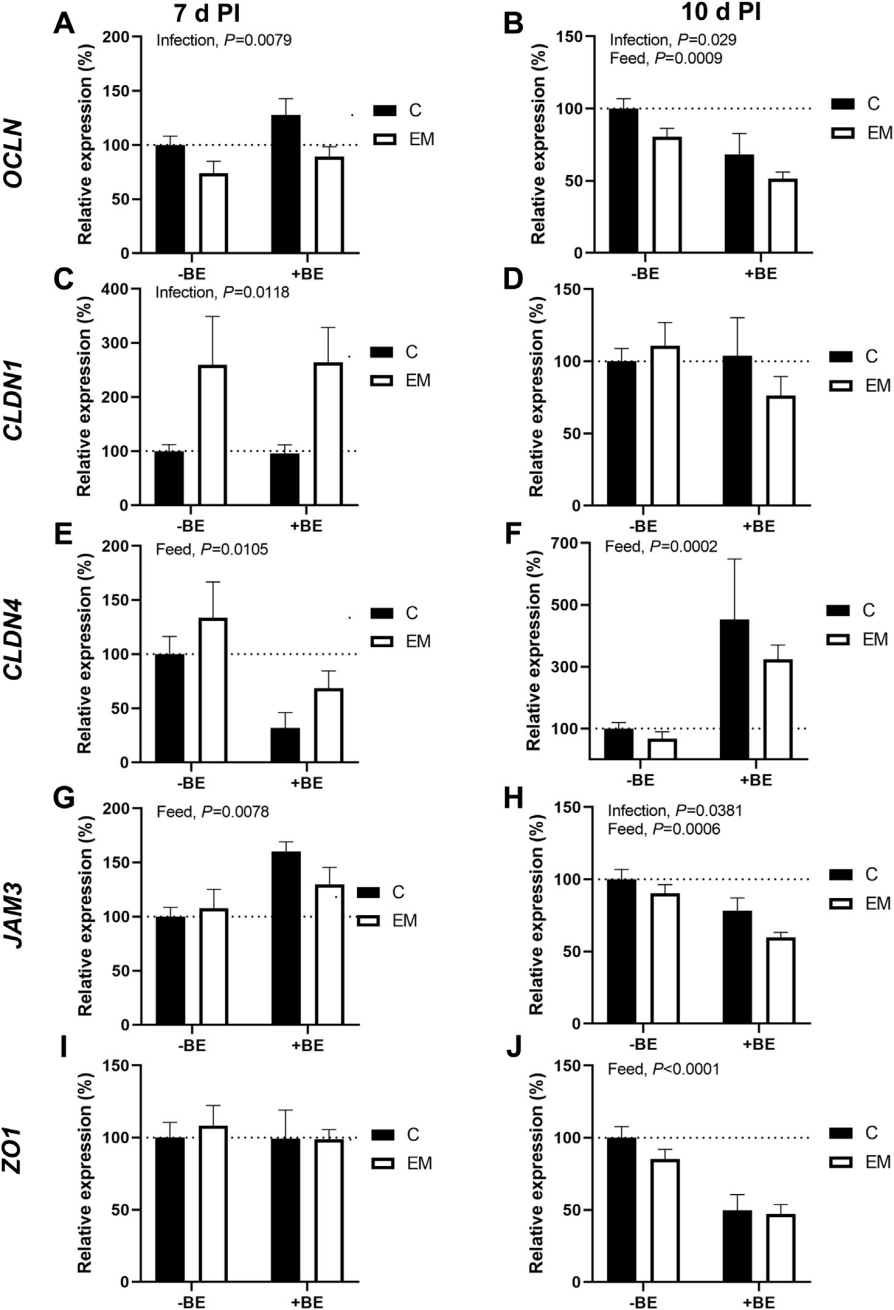
Effect of presence (+BE) or absence (−BE) of tributyrin in a feed for control (C) or *E. maxima* (EM)–challenged birds on *OCLN*, *CLDN1*, *CLDN4*, *JAM3*, and *ZO1* mRNA level in chicken ileum at either 7 (7 d PI) or 10 d postinfection (10 d PI). The expression level of C-BE was set to 100% (dotted line), and the other values are presented as % of the C-BE data. Each value represents mean ± SE (n = 6). Abbreviations: *CLDN1*, claudin 1; *CLDN4*, claudin 4; *JAM3,* junctional adhesion molecule 3; *ZO1*, zona occludens 1.

The mRNA levels of *FABP2* were significantly impacted by infection (*P* < 0.0001). Broiler chickens in the EM groups had lower *FABP2* mRNA levels than C groups (Figure 4E). The *FABP2* mRNA levels at 10 d PI were similarly affected by infection (*P* = 0.0012) with lower levels in the EM groups (Figure 3F).

For *MUC2* at 7 d PI, there was not a significant impact of either infection or feed and mRNA levels in test groups were only slightly lower than 100% (Figure 3G). However, at 10 d PI, there was a significant impact of feed (*P* = 0.0003) on *MUC2* mRNA levels. The expression of MUC2 was lower in +BE groups at 10 d PI (Figure 3H).

### Tight Junction Genes

The relative mRNA expression of tight junction genes occludin (***OCLN***), claudin 1 (***CLDN1***), claudin 4 (***CLDN4***), junctional adhesion molecule 3 (***JAM3***), and zona occludens 1 (***ZO1***) can be found in Figure 4. Junctional adhesion molecule 2 was also tested, but no significant differences between groups were found so those data are omitted here (data not shown).

Relative mRNA levels of *OCLN* at 7 d PI was significantly affected by infection (*P* = 0.0079) with EM groups having lower *OCLN* levels than C groups (Figure 4A). At 10 d PI, *OCLN* mRNA levels were significantly affected by both infection (*P* = 0.029) and feed (*P* = 0.0009). Overall, at 10 d PI, *OCLN* mRNA levels in EM groups were lower than those in C groups, and +BE groups were lower than -BE groups (Figure 4B).

The mRNA levels of *CLDN1* at 7 d PI were significantly affected by infection (*P* = 0.0118). The EM groups had higher *CLDN1* mRNA levels than C groups (Figure 4C). On 10 d PI, *CLDN1* was not affected by either infection or feed (Figure 4D).

For *CLDN4* at 7 d PI, there was a significant impact of feed (*P* = 0.0105) on mRNA levels. The mRNA levels of *CLDN4* were lower in +BE groups at 7 d PI (Figure 4E). At 10 d PI, while the *CLDN4* mRNA levels were also significantly impacted by feed (*P* = 0.0002), the +BE groups instead had higher mRNA levels than the ones on normal feed (Figure 4F).

The mRNA levels of *JAM3* at 7 d PI were significantly affected by feed (*P* = 0.0078) with higher *JAM3* levels in +BE groups (Figure 4G). At 10 d PI, the *JAM3* mRNA levels were significantly affected by both infection (*P* = 0.0381) and feed (*P* = 0.0006). Overall, at 10 d PI, *JAM3* mRNA levels in EM groups were lower than those in C groups, and +BE groups were lower than -BE groups (Figure 4H).

For *ZO1* at 7 d PI, there were no significant effects of either infection or feed, and the mRNA levels of all groups were similar (Figure 4I). At 10 d PI, *ZO1* mRNA levels were significantly affected by feed (*P* < 0.0001), and +BE groups had lower mRNA levels than normal feed ones (Figure 4J).

## Discussion

Chickens challenged with *E. maxima* were characterized by lower BW at 7 d PI. Similar results were observed by others (Wu et al., 2014; Kim et al., 2017; Miska and Fetterer, 2017; Su et al., 2018; Fortuoso et al., 2019). Addition of butyrate glycerol esters to poultry feed ameliorated the negative effect of infection on BWG. Similar effects were seen by Ali et al. (2014) in chicks infected with *E. maxima*. Remarkably, significant attenuation of negative effects of *Eimeria* infection on BWG by addition of BE to the diet was observed with only 3 replicates per treatment. Many studies have reported the positive effect of butyrate on broilers BW in healthy birds (Smulikowska et al., 2009; Ali et al., 2014; Levy et al., 2015; Liu et al., 2017; Sikandar et al., 2017). In our study, we did not observe increased BWG in chickens receiving only BE. This is in agreement with Leeson et al. (2005), Zhang et al. (2011), and Smulikowska et al. (2009). The decrease in BWG during coccidia challenge is likely because of damage of intestinal epithelial cells that results in a decrease in expression of digestive enzymes and nutrient transporters and possibly a decrease in the ability of the broilers to take up available nutrients. Changes in the expression profile of several digestive enzymes and nutrient transporters in birds challenged with *Eimeria* have been reported (Fetterer et al., 2014; Su et al., 2015, 2018; Miska and Fetterer, 2017, 2018). Recently, Fortuoso et al. (2019) showed that coccidiosis causes an alteration in carbohydrate and lipid metabolism that can contribute to impaired animal performance. Moreover, these researchers have shown that activation of serum enzymes linked to ATP synthesis and utilization can be a potential pathway involved in the reduction of BW in *Eimeria* challenged broilers (Fortuoso et al., 2019). Butyrate has also been shown to play a role in the infection control of other pathogens such as *Salmonella enterica* and *Clostridium perfringens* (Van Immerseel et al., 2005; Fernandez-Rubio et al., 2009). Moreover, butyrate has been reported to have antiinflammatory properties and to be involved in inhibiting nuclear factor-kappa B activation, followed by decreased expression of proinflammatory cytokines (Inan et al., 2000; Place et al., 2005), probably resulting in saving energy for growth.

The decrease in BWG was accompanied by no change in FI during coccidia challenge regardless of the presence of butyrate. However, FCR was significantly increased in *E. maxima–*challenged birds that were not receiving BE 7 d PI. At 10 d PI, FCR was increased in infected birds but not when the feed was treated with BE. In contrast to our results, Wu et al. (2014) and Ali et al. (2014) observed increased FI during coccidia challenge, whereas Bozkurt et al. (2014) showed a decrease in feed intake during coccidia challenge. On the other hand, similar to our results, they also observed elevated FCR during coccidia infection (Ali et al., 2014; Bozkurt et al., 2014; Wu et al., 2014). Elevated FCR 10 d PI may reflect compensatory FI to catch up with growth requirement because numerically, BW was lower, and FI was minimally higher in infected birds at day 10 PI. Some of the discrepancies between these results could be explained by the age of experimental birds, dose of the *E. maxima* used for the challenge, and the pathogenicity of the strain. In our study, we did not observe a positive effect of BE supplementation on FI and FCR in healthy birds. Similar results in healthy birds were observed by Smulikowska et al. (2009). In contrast to our results, Ali et al. (2014) reported decreased FI and lower FCR in healthy birds that were supplemented with BE and ameliorating effects of BE in birds infected with coccidia. There are many forms of butyrate that could be used for feed supplementation in birds (Leeson et al., 2005; Smulikowska et al., 2009; Ali et al., 2014; Levy et al., 2015; Sikandar et al., 2017), and differences between them could be one of the reasons for different results in BWG, FI, and FCR. For this study, we fed 5 to 10 times the recommended dose by Perstorp for starter, grower, and finisher phases for broiler chickens and that may have an effect as well.

A decrease in plasma carotenoids was observed during *E. maxima* infection, but the presence of BE did not influence this parameter. Even though BE addition ameliorated the negative effect of coccidia infection on BWG, its mechanism does not appear to function by attenuation of intestinal damage because carotenoid levels in infected birds treated with BE were similar to those in birds not supplemented with BE.

To shed some light on the origins of any observed effects of *E. maxima* and tributyrin supplementation on birds, we also measured mRNA levels of targeted genes in the ileum. For this study, we examined a collection of immune, digestion, and gut integrity genes that might be affected by either *E. maxima* inoculation or tributyrin in feed. We compared the 3 test groups to the control noninfected birds receiving normal feed and looked for differences in mRNA levels.

Liver-enriched antimicrobial peptide 2 is a cationic antimicrobial peptide that has been shown to have activity against a broad range of Gram-positive bacteria (Townes et al., 2009). Here, we observed that *LEAP2* transcription was significantly decreased for infected samples at 7 d PI, regardless of whether the chickens were being fed tributyrin or not. This was expected given that previous reports of LEAP2 expression data from gut tissues have also shown decreased expression during *Eimeria spp*. infection (Casterlow et al., 2011; Sumners et al., 2011; Fetterer et al., 2014; Su et al., 2015, 2017). It has been proposed that *LEAP2* expression is decreased during *E. spp.* infection because of the parasite modulating the transcription of the chicken host cells (Paris and Wong, 2013). Another study observed *LEAP2* transcription during *Eimeria* infection in a supposedly coccidiosis-resistant Fayoumi chicken line similar to noninfected levels of transcription (Su et al., 2018). However, the “resistant” Fayoumi line experienced similar loss of weight gain to the nonresistant line (Su et al., 2018). Therefore, simply increasing *LEAP2* transcription during *E. maxima* infection in broilers would be unlikely to rescue loss of weight gain. Interestingly at 10 d PI, there was no longer significant suppression of *LEAP2* mRNA levels for EM birds. If *E. maxima* does downregulate the expression of *LEAP2,* then this would make sense because at 10 d PI, the chicken is shedding approximately 1% of the number of oocysts it was at 7 d PI (Allen at al., 2005; Jenkins et al., 2017). Su et al. (2018) demonstrated that *LEAP2* is expressed in epithelial cells of the villi in jejunal tissues at 7 d PI in both *E. maxima*-infected Ross broilers and controls. However, they also showed that the villi of the infected birds were significantly shorter which might explain the reduced *LEAP2* transcription in the infected birds (Su et al., 2018). Even though the effect was not significant, there was a trend in *LEAP2* to be lower at 10 d PI in birds feed tributyrin. It would be interesting to examine the localization of *LEAP2* in the epithelium of chicken intestinal tissues during the recovery period compared with the height of infection. Observing which cells express LEAP2, both mRNA and protein, and at what levels during recovery may lead to more insights about the role of LEAP2 during *Eimeria* infections in chickens.

Interleukin-1 beta is a proinflammatory cytokine that varies in transcription and protein expression during infection depending on the pathogen (Giansanti et al., 2006). We observed at 7 d PI, there was a significant effect of the interaction of infection status and feed type on *IL1B* mRNA levels. Although Tukey's test of multiple comparisons did not find significant differences between groups (data not shown), analysis of data suggest that either *IL1B* mRNA levels are decreased in EM birds but only without tributyrin supplementation, or +BE birds did have lower *IL1B* but only in noninfected groups. In contrast, Tan et al. (2014) observed an increase in *IL1B* mRNA expression in jejunums of broilers after 7 d postinoculation with a live attenuated vaccine of 4 *E. spp*. including *E. maxima*. This may be because of the differences in pathogenicity of the *E. maxima* strains employed, the addition of other *E. spp.*, or the sample tissue types. While there were not significant effects of tributyrin supplementation on *IL1B* mRNA levels, there was a trend observed at 10 d PI for +BE groups to have higher expression regardless of infection status. Because we observed a similar but opposite trend in *LEAP2* that may be an indication that butyrate is having additional effects on immune gene regulation. It may be beneficial to examine immune cell activity in butyrate feeding studies in the future.

Aminopeptidase N is an enzyme that cleaves amino acids from the N-terminus, and it is present in many different organs, and in the chicken intestine, it is located at the brush border (Luan and Xu, 2007; Miska et al., 2015). In a previous study, decreased *APN* transcription was observed in broiler jejunum after *E. maxima* infection through 14 d PI, though it was only significantly lower at 7 d PI (Miska and Fetterer, 2018). In the present study, we observed significantly lower *APN* mRNA levels in all EM groups at 7 d PI but not 10 d PI which is in accordance with Miska and Fetterer (2018). In the present study, at 10 d PI, there was a significant effect of feed type where +BE groups had lower *APN* mRNA levels. This is not unexpected given that diet composition has previously been shown to have effects on *APN* mRNA levels in broiler chickens, particularly in the ileum (Gilbert et al., 2010).

Monocarboxylate transporter 1 is a monocarboxylate transporter of gut microflora metabolites in the intestine, including butyrate (Tamai et al., 1995; Kirat et al., 2006). Previously butyrate supplementation has been shown to upregulate both MCT1 mRNA and protein in human colonic epithelial cells (Cuff et al., 2002). However, tributyrin supplementation did not appear to have an effect on *MCT1* expression in the present study. This is in contrast to a previous study in pigs, where butyrate supplementation was shown to increase *MCT1* transcription in the ileum, the site where their utilized form of butyrate was released by encapsulation (Lacorn et al., 2010). We observed that *E. maxima* infected birds had increased *MCT1* mRNA levels at 7 d PI. It would be interesting to examine further if the upregulation of *MCT1* during coccidiosis is a regulatory effect by the *E. maxima* infection or a host response to the infection.

Fatty-acid binding protein 2 is a lipid-binding transporter that acts as an important regulator of fat uptake in the chicken intestine (Wang et al., 2005; Hu et al., 2010). We observed that *FABP2* mRNA levels were significantly lower in EM groups on both 7 and 10 d PI, though the effect was more pronounced at 7 d PI. This was an expected result given that low expression of FABP2 is an indicator of damaged epithelium in the bird gut (Chen et al., 2015). We also did not observe any significant effects of tributyrin supplementation on *FABP2* transcription. Other recently examined dietary supplements in broilers, such as olive pomace extract and phytogenic premix, also have not had a significant effect on *FABP2* transcription in healthy broilers (Mountzouris et al., 2019; Herrero-Encinas et al., 2020).

Mucin 2 is a polymerizing protein excreted by intestinal goblet cells that helps to form a mucosal barrier between intestinal epithelium and the lumen of the gut of chickens (Smirnov et al., 2004; Horn et al., 2009). It was surprising that infection status did not have a significant effect on *MUC2* mRNA levels at 7 or 10 d PI because other studies have found reduced MUC2 expression in the intestine during *Eimeria* infection (Tan et al., 2014; Chen et al., 2015). However, *MUC2* mRNA levels were lower in +BE groups at 10 d PI. Song et al. (2017) observed significantly increased *MUC2* expression in chickens supplemented with sodium butyrate during necrotic enteritis (**NE**) induced by *E. spp* and *C. perfringens*. However, they did not include an uninfected sodium butyrate fed control group so the effects of pathogen and feed type are difficult to distinguish.

Alterations in tight junctions can cause modifications in gut permeability in the intestinal epithelium (Awad et al., 2017). Therefore, we expected to see a similar pattern in expression of tight junction genes during infection with just *E. maxima*. However, this was not the case in our study, where diverse results for *OCLN*, *CLDN1*, *CLDN4*, *JAM3*, and *ZO1* expression were observed. Occludin is an important component of tight junctions in the chicken intestine (Furuse et al., 1993; Chen et al., 2015). In the case of *OCLN*, mRNA levels were decreased in EM groups at both 7 and 10 d PI. This indicates a decrease in the integrity of the gut barrier and has been observed previously in broilers during *E. maxima* infection (Al-Sadi et al., 2011; Song et al., 2017; Leung et al., 2019). At 10 d PI, the +BE groups also had significantly decreased *OCLN* mRNA levels. Though *OCLN* expression has been previously shown to be rescued by butyrate supplementation during NE, we did not observe such an effect on solely *E. maxima* infected birds (Song et al., 2017).

Claudin 1 and CLDN4 are components of tight junctions that are expressed in the chicken intestinal epithelium (Simard et al., 2005). At 7 d PI, there were significantly higher mRNA levels for *CLDN1* in EM groups, and this is consistent with what Castro e al. (2020) observed during infection with multiple *Eimeria spp*. including *E. maxima*. We observed *CLDN4* mRNA levels to be significantly affected by tributyrin supplementation at both 7 and 10 d PI. Interestingly, the effect seemed to be opposite at the 2 time points, with *CLDN4* being down in +BE groups at 7 d PI and up at 10 d PI. Though *CLDN4* levels have been previously observed to be increased with a sodium butyrate diet during NE, the effect of butyrate alone was not investigated (Song et al., 2017).

Junctional adhesion molecule 3 and ZO1 are additional tight junction proteins that help to maintain the gut barrier (Tsukita et al., 2001). The *JAM3* mRNA levels were affected by tributyrin supplementation differentially at 7 and 10 d PI with increased expression in +BE groups at 7 d PI and decreased expression at 10 d PI. *E. maxima* infection also decreased JAM3 expression at 10 d PI. The mRNA levels of *ZO1* did not appear to be affected by either infection or feed type at 7 d PI, but at 10 d PI, the +BE groups had lower *ZO1* expression. Chen et al. (2015) did not find *JAM3* or *ZO1* expression to be significantly affected in a model of “gut barrier failure” in chickens inoculated with *E. maxima* along with other *Eimeria spp*. to induce coccidiosis. Therefore, it was not surprising that these genes were not significantly impacted by infection at 7 d PI. While the effect of tributyrin supplementation on these tight junction genes is unclear for now, additional studies on the direct effects of butyrate would be illuminating in the future.

Because different breeds of chickens have been shown to have different levels of susceptibility to *Eimeria spp*., the gene transcription results we observed in Ross 708 broilers may not be the same to what would be measured in other strains of birds (Long, 1968; Bumstead and Millard, 1987). From the results reported here, tributyrin could have the potential to improve the performance of Ross 708 broilers during *E. maxima* infection, and its use should be studied in birds infected with other *Eimeria* species. However, further studies into the physiopathological effects of *Eimeria spp*. should be conducted to better understand promising gene targets and feed additives that could work upon them.
